# Case of Optical Illusion from Hysteria

**Published:** 1805-08-01

**Authors:** T. Bishopp

**Affiliations:** Leicester


### Case of Optical Illusion from Hysteria; *communicated by Mr.* T. Bishopp, *of Leicester.*

A Single woman, of delicate frame, aged 22, had been much afflicted with Hysteria more than three months. The paroxysms of the disease were often violent, accompanied frequently, but not constantly, with temporary delirium: so that the disease appeared to be well marked, never being preceded by any local irritation, of which the patient was conscious. During some of these attacks she was occasionally so much in possession of the faculties of the mind and of speech, as to be able to reply appositely to questions put to her by the attendants; but of these conversations she retained no recollection whatever upon the termination of the paroxysm. Certain paroxysms were productive of convulsions so violent as to require coercion; while others were attended merely with mild delirium. In the latter, impressions made by surrounding objects upon the retina, were transmitted to the brain, as usual inverted, and were represented to the mind in that position so forcibly, that the young woman could not resist the impulse she felt to place the chairs in the room horizonhorizontally, lest they should fall, finding they would not stand on the other end. She expressed her surprise, and laughed heartily, on seeing the attendants all standing, as she thought, upon their heads. The illusion immediately subsided with the fit, both lasting about an hour generally. This, therefore, was not a singular occurrence in one particular fit, but recurred repeatedly. The whole disease yielded, at length, under my care, to the ordinary treatment of hysteria, no defect either in the organ of vision, or in the faculties of the mind, remaining.

